# High anemia prevalence in Korean older adults, an advent healthcare problem: 2007–2016 KNHANES

**DOI:** 10.1186/s12877-020-01918-9

**Published:** 2020-11-26

**Authors:** Hee Won Chueh, Hye Lim Jung, Ye Jee Shim, Hyoung Soo Choi, Jin Yeong Han, on the behalf of the Red Blood Cell Disorder Working Party of The Korean Society of Hematology

**Affiliations:** 1 Department of Pediatrics, Dong-A University College of Medicine, Busan, Republic of Korea; 2 Department of Pediatrics, Sungkyunkwan University School of Medicine, Seoul, Republic of Korea; 3 Department of Pediatrics, Keimyung University School of Medicine, Keimyung University Dongsan Hospital, Daegu, Republic of Korea; 4 Department of Pediatrics, Seoul National University Bundang Hospital, Seoul National University College of Medicine, Seongnam, Republic of Korea; 5 Department of Laboratory Medicine, Dong-A University College of Medicine, 26 Daesingongwon-ro, Seo-gu, Busan, 49201 Republic of Korea; 6 The Korean Society of Hematology, Seoul, Republic of Korea

**Keywords:** Anemia, Older adults, KNHANES, Comorbidity

## Abstract

**Background:**

Anemia is associated with high morbidity and mortality in older people. However, the prevalence and characteristics of anemia in older individuals are not fully understood, and national data on these aspects in older Korean adults are lacking. This study aimed to evaluate the prevalence and characteristics of anemia in older adults using data from the Korea National Health and Nutrition Examination Survey (KNHANES), which is a nationwide cross-sectional epidemiological study conducted by the Korean Ministry of Health and Welfare.

**Methods:**

Data from a total of 62,825 participants of the 2007–2016 KNHANES were compiled and analyzed to investigate differences in participant characteristics and potential risk factors for anemia. Differences in clinical characteristics of participants were compared across subgroups using the chi-square test for categorical variables and independent *t*-test for continuous variables. Univariate and multivariate analyses using logistic regression were performed to identify related clinical factors.

**Results:**

The prevalence of anemia was higher in the population aged ≥65 years than in the younger population. Anemia was also more prevalent among females than among males, but this difference was not significant in people aged > 85 years. Being underweight, receiving a social allowance, living alone, and having comorbidities such as hypertension, rheumatoid arthritis, diabetes mellitus (DM), cancer, and chronic renal failure (CRF) were more common among older adults with anemia than among the population without anemia. In univariate and multivariate analyses, older age, female sex, underweight, and presence of comorbidities including rheumatoid arthritis, DM, cancer, and CRF were associated with an increased risk of anemia.

**Conclusions:**

This study revealed that age, female sex, underweight, and the presence of comorbidities such as rheumatoid arthritis, DM, cancer, and CRF were associated with an increased risk of anemia in older Korean adults. Further study on causal relationships between anemia and other variables in the older population is necessary.

## Background

The prevalence of anemia is known to increase with age. A previous epidemiological study in the U.S. showed that anemia was prevalent in > 10% of people aged ≥65 years (older adults) and in > 20% of those aged ≥85 years [1]. According to the 2018 National Health Statistics for Korea, the prevalence of anemia in men and women aged 60–69 years was 5.3 and 8.0%, respectively, and in those aged > 70 years, the prevalence was higher, at 16.4 and 18.3%, respectively [2]. Anemia in older people has been reported to be more prevalent in individuals who are residents of nursing homes and those who are hospitalized [3–6].

Anemia in the older population is reported to be related to various medical burdens, including increased morbidity and mortality [7]. In particular, anemia is reported to be related to higher cardiovascular events [8, 9], frailty [3, 10, 11], fractures [12], prolonged hospital stays, and unfavorable outcomes [13–15]. Robins et al. showed that anemia in older population possesses potential morbidity and mortality by affecting tissue oxygen delivery, accompanying the aging process, and eventually leading to multiple major and minor organ dysfunction, affecting both physical and mental functions [16]. Increasing prevalence of anemia in the older population will potentially increase the associated medical cost and social burden.

However, anemia in older people is not fully understood. Anemia in older adults is reported to be mainly due to nutritional deficiency following chronic kidney disease, blood cell disorders, malignancy, and other diseases [5, 17, 18]. However, the etiology of anemia is unclear in a certain proportion of individuals despite various tests being conducted in this group, including bone marrow aspiration and biopsy [1, 19, 20].

The population of older people in many developing and developed countries, including South Korea, is proportionally increasing. In South Korea, the average life expectancy is increasing, while the birth rate per woman is decreasing. According to Statistics Korea, the proportion of population aged 65 years and older in South Korea was 14.3% in 2018 and is predicted to increase to 46.5% by 2067 [21]. Despite these findings, the social and health policies and provisions for older people in Korea are inadequate. However, there are few studies that reported anemia in older adults of Korea. These reports are limited to single-center or small community cross-sectional studies, and do not represent the current status of the Korean population. In this regard, this study aimed to evaluate the actual prevalence of anemia in older adults in South Korea and elucidate the burden of health problems related to anemia, using data from a large nationwide survey.

## Methods

### Study population and characteristics

This study used data from the Korea National Health and Nutrition Examination Survey (KNHANES). The KNHANES is a nationwide health and nutrition survey designed by Korea Centers for Disease Control & Prevention, and conducted annually by the South Korean government, according to the Article 16 of National Health Promotion Act. The survey was first conducted in 1998, and its structure and format have substantially evolved since 2007. The survey includes questionnaires and details of laboratory tests, which have been regularly revised every 3 years. However, there have been considerable changes since 2007 in major formats of the survey. The sampling method has changed from short-term cross-sectional sampling to circular sampling, which is a continuous method. Central laboratory centers, several methodologic formats of blood tests, and data-input formats have also undergone revisions and changes. Therefore, for reasons concerning data homogeneity, this study compiled and analyzed data from the KNHANES conducted from 2007 to 2016.

The participants of the KNHANES include community-dwelling Koreans, carefully selected by statistical methods that use multivariable stratification and cluster sampling to represent the entire Korean population. The survey excludes individuals from military troops, prisons, hospitals, and nursery or social homes. Participants are selected and enrolled as family units to gather complete data on all family members. Written informed consent is obtained from the participants at the start of each survey examination. Personal data and results of the survey are de-identified before they are made publicly available. As an annually conducted cross-sectional study, no individual or family is allowed to participate in the KNHANES repeatedly.

KNHANES examination study was approved by the Korean Centers for Disease Control & Prevention Research Ethics Review Committee, which is renewed annually. Ethical approval of this study was obtained through the Institutional Review Board of Dong-A University Hospital.

### Definition of terms

Anemia was defined as a hemoglobin level of < 13.0 g/dL for men and < 12.0 g/dL for women, based on the World Health Organization criteria for anemia. Being underweight was defined as a body mass index (BMI) of < 18 kg/m^2^. Social allowance was defined as one or one’s family being the beneficiary of social allowance, which is run as a single system by the Korean government. Household income was initially classified into four categories in KNHANES, and the lowest household income was defined as “low household income.” Under the item “food insecurity,” “insecurity” was defined as having the following answer to the questionnaire: “have trouble often or frequently in having adequate amount and quality of daily food for economic reasons.” Because KNHANES enrolled the participants as a family unit, “live alone” was defined as a family consisting of a single family member. Comorbid conditions were defined as those diagnosed by a doctor, as reported by the participants. Hypertension referred to a resting systolic blood pressure of > 140 mmHg or a diastolic blood pressure of > 90 mmHg at examination. Use of antihypertensive medication was also included to the hypertension group though their blood pressure checked on examination site were within the normal limit. According to the KNHANES, to reduce the variation, they adopted certification in measuring blood pressure, and educated the personnel to measure blood pressure, employing a uniform method. Diabetes mellitus (DM) was defined as having been diagnosed with DM or treated for DM or having a fasting blood glucose level of > 126 mg/dL. Osteoarthritis, rheumatoid arthritis, asthma, hepatitis B, hepatitis C, and liver cirrhosis were defined as the respective conditions having been diagnosed by a doctor or the individual currently being affected by the disease or having undergone treatment for the conditions. Pulmonary tuberculosis was defined as an individual having been diagnosed with tuberculosis, or currently affected by from it, or use of anti-tuberculosis medication, including subclinical tuberculosis. Cancer was defined as an individual currently having a diagnosis and treatment of any cancer type. Chronic renal failure (CRF) was defined as an estimated glomerular filtration rate of < 60 mL/min, using the Modification of Diet in Renal Disease Study equation.

### Statistical analysis

The KNHANES has specific guidelines for statistical analysis, including cluster sampling and stratification, to ensure the accuracy of data [22]. To represent the entire Korean population, the sampling weights assigned to the participants were applied to all analyses and were generated considering the complex sample design, nonresponse rate of the target population, and post-stratification. Differences in participant characteristics were compared across subgroups using the chi-square test for categorical variables and the independent *t*-test for continuous variables, as appropriate. Trend analysis was performed for a complex survey design using logistic regression analysis. Univariate and multivariate analyses using logistic regression were performed to identify prognostic factors that were independently related to anemia in older adults. As hemoglobin alone can be used to show significant differences between participants with and without anemia, this variable was removed during the multivariate analysis. The prevalence of anemia was plotted by age and sex for data visualization. All statistical analyses were carried out using IBM SPSS® Statistics version 24.0 (IBM, Armonk, NY). A two-sided *P*-value of < 0.05 was considered statistically significant.

## Results

**Fig. 1:**
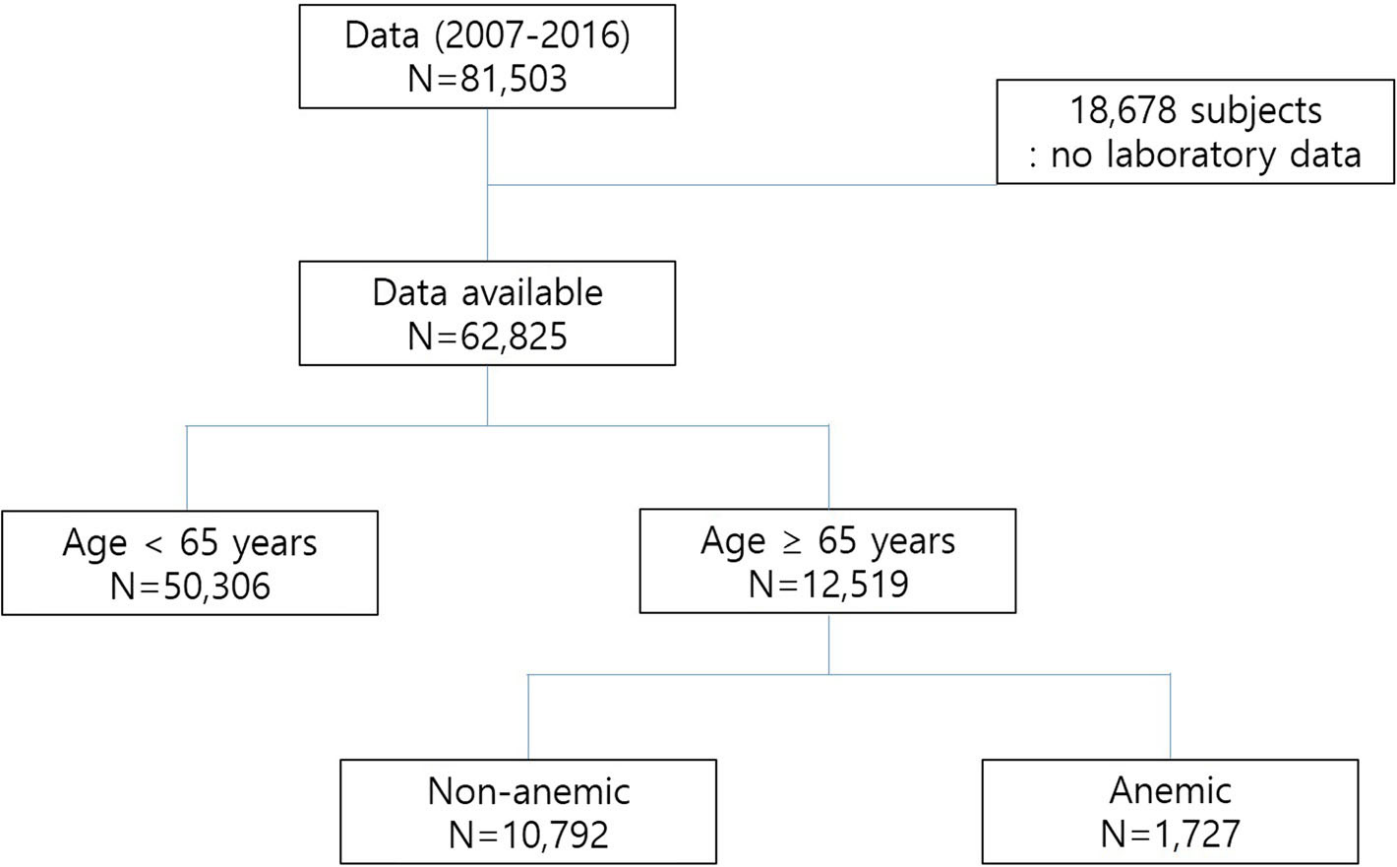
Flowchart of the study patient selection process. Data from the 2007–2016 Korea National Health and Nutrition Examination Survey (KNHANES)

**Fig. 2:**
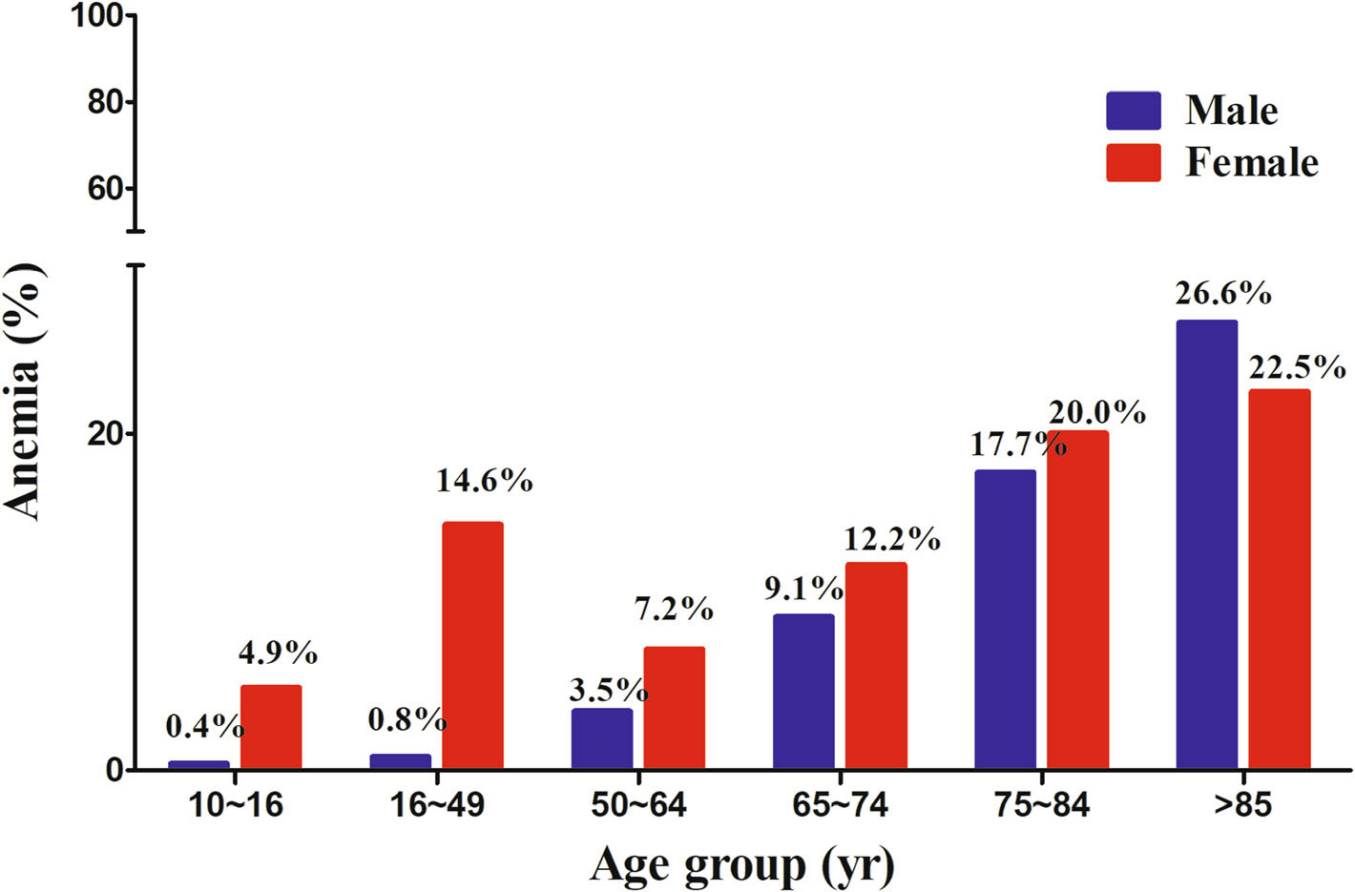
Prevalence of anemia according to age group

The data of 81,503 participants in the 2007–2016 KNHANES were retrieved. Among the participants, 18,678 were excluded from this study because of missing laboratory data. Thus, a total of 62,825 participants were included in the analysis, of whom 12,519 were aged ≥65 years (Fig. 1). Comparison of basic clinical characteristics between the groups with and without laboratory data in the population aged 65 years and older are shown in Table 1. Most of these basic clinical characteristics showed significant differences between the two groups. The age distribution was analyzed according to the survey periods. A trend of increasing proportion of older adults was observed, although with an insignificant difference (Fig. 2).

**Table 1:** Comparison of baseline characteristics of patients with and without laboratory data

Variable	Older adults with laboratory data	Older adults without laboratory data	*P*-Value
	(*n* = 5,143,890.215)^c^	(*n* = 830,027.379)^c^	
Mean age, years	72.24 ± 0.06	76.02 ± 0.17	< 0.001^a^
Women, %	56.0 ± 0.5	65.9 ± 1.3	< 0.001^b^
Underweight (BMI < 18), %	2.3 ± 0.2	5.8 ± 0.7	< 0.001^b^
Social allowance, %	11.0 ± 0.4	15.0 ± 1.0	< 0.001^b^
Low household income, %	48.8 ± 0.7	57.5 ± 1.5	< 0.001^b^
Food insecurity	2.1 ± 0.2	2.9 ± 0.4	0.034^b^
Live alone, %	16.2 ± 0.4	21.2 ± 1.1	< 0.001^b^
Condition, %			
Hypertension	62.7 ± 0.5	65.1 ± 1.5	0.130^b^
Osteoarthritis	30.5 ± 0.5	30.6 ± 1.4	0.913^b^
Rheumatoid arthritis	4.1 ± 0.2	4.1 ± 0.6	0.936^b^
DM	23.6 ± 0.5	88.7 ± 1.8	< 0.001^b^
CVD	6.1 ± 0.2	7.9 ± 0.9	0.034^b^
Asthma	5.5 ± 0.3	6.2 ± 0.8	0.367^b^
Pulmonary tuberculosis	0.2 ± 0.0	0.4 ± 0.2	0.259^b^
Stroke	4.3 ± 0.2	6.0 ± 0.7	0.007^b^
Cancer, current	2.2 ± 0.2	6.4 ± 0.7	< 0.001^b^
Hepatitis B	1.2 ± 0.1	0.8 ± 0.2	0.145^b^
Hepatitis C	0.2 ± 0.0	0.6 ± 0.2	0.014^b^
Liver cirrhosis	0.6 ± 0.1	0.4 ± 0.2	0.359^b^
CRF	16.9 ± 0.4	31.8 ± 5.2	< 0.001^b^

Values are expressed as either percentage ± standard error (SE) of the percentage or mean ± SE

*Abbreviations*: *BMI* body mass index, *DM* Diabetes mellitus, *CVD* Cardiovascular disease, *CRF* Chronic renal failure

^a^*P*-values were derived from the independent *t*-test

^b^*P*-values were derived from the chi-squared test

^c^Weighted population

### Prevalence of anemia and its trend

Of the 62,825 participants, 5315 had anemia, including 1024 males and 4291 females. The overall prevalence of anemia in the population aged ≥10 years was 7.3% (95% confidence interval [CI], 7.1–7.5%). The prevalence of anemia in the population aged ≥65 years was 14.0% (95% CI, 13.3–14.7%), whereas that in the population aged < 65 years was 6.4% (95% CI, 6.2–6.6%). The prevalence of anemia was higher in females (12.2%; 95% CI, 11.8–12.6%) than in males (2.5%; 95% CI, 2.3–2.7%). However, in the population aged > 85 years, the prevalence of anemia between men and women did not show any significant difference (26.6% vs. 22.5%, *P* = 0.577; Fig. 2).

The trend of anemia was analyzed through logistic regression. A significant increase was observed for overall (*P* < 0.001), male (*P* < 0.001), and female (*P* < 0.001) populations.

### Difference in characteristics between older adults with and without anemia

The baseline characteristics of older adults with and without anemia are shown in Table 2. Known risk factors for anemia were included as variables in the analysis. The population with anemia had a higher proportion of older adults and women. The group with anemia also showed a higher proportion of participants who were underweight, were beneficiaries of social allowance, and who were living alone, than the group without anemia. No significant differences in household income or food insecurity were observed. Moreover, the population with anemia tended to have a higher prevalence of hypertension, osteoarthritis, rheumatoid arthritis, DM, cancer, and CRF (Table 2).

**Table 2:** Comparison of baseline characteristics of older adults with and without anemia

Variable	Older adults without anemia	Older adults with anemia	*P*-Value
	(*n* = 4,444,812.928)^c^	(*n* = 699,077.287)^c^	
Mean age, years	71.96 ± 0.06	74.00 ± 0.15	< 0.001^a^
Women, %	55.1 ± 0.5	61.8 ± 1.3	< 0.001^b^
Mean Hb level (SE)	14.00 ± 0.01	11.48 ± 0.03	< 0.001^b^
Underweight (BMI < 18 kg/m^2^), %	2.0 ± 0.2	4.5 ± 0.6	< 0.001^b^
Social allowance, %	10.6 ± 0.4	13.5 ± 1.0	0.002^b^
Low household income, %	48.5 ± 0.7	50.4 ± 1.4	0.200^b^
Food insecurity	2.0 ± 0.2	2.2 ± 0.4	0.626^b^
Live alone, %	15.8 ± 0.4	19.0 ± 1.0	0.001^b^
Condition, %			
Hypertension	62.3 ± 0.6	65.3 ± 1.3	0.034^b^
Osteoarthritis	30.0 ± 0.5	33.7 ± 1.4	0.010^b^
Rheumatoid arthritis	3.8 ± 0.2	6.1 ± 0.6	< 0.001^b^
DM	22.5 ± 0.5	30.7 ± 1.4	< 0.001^b^
Asthma	5.6 ± 0.3	5.1 ± 0.6	0.406^b^
Pulmonary tuberculosis	0.2 ± 0.1	0.2 ± 0.1	0.578^b^
Cancer, current	1.9 ± 0.2	4.4 ± 0.6	< 0.001^b^
Hepatitis B	1.3 ± 0.1	0.9 ± 0.2	0.156 ^b^
Hepatitis C	0.3 ± 0.0	0.2 ± 0.1	0.669 ^b^
Liver cirrhosis	0.6 ± 0.1	0.7 ± 0.2	0.701^b^
CRF	14.2 ± 0.5	34.0 ± 1.2	< 0.001^b^

Values are expressed as either percentage ± standard error (SE) of the percentage or mean ± SE

*Abbreviations*: *Hb* hemoglobin, *BMI* body mass index, *DM* Diabetes mellitus, *CRF* Chronic renal failure

^a^*P*-values were derived from the independent *t*-test

^b^*P*-values were derived from the chi-square test

^c^Weighted population

Relationships between independent and dependent variables were analyzed using univariate and multivariate logistic regression. Analysis of risk factors for anemia in older adults is shown in Table 3. Mean hemoglobin levels were not included in further analysis due to the definite differences in hemoglobin levels between participants with and without anemia. Based on the results, the risk of anemia increased with older age (odds ratio [OR], 1.058; 95% CI, 1.045–1.070; *P* < 0.001) and with female sex (OR, 1.252; 95% CI, 1.080–1.453; *P* = 0.001). Being underweight (OR, 2.197; 95% CI, 1.570–3.075; *P* < 0.001) and presence of rheumatoid arthritis (OR, 1.436; 95% CI, 1.109–1.860; *P* = 0.006), DM (OR, 1.438; 95% CI, 1.248–1.656; *P* < 0.001), cancer (OR, 2.675; 95% CI, 1.890–3.786; *P* < 0.001), and CRF (OR, 2.557; 95% CI, 2.212–2.955; *P* < 0.001) were also associated with an increased risk of anemia.

**Table 3:** Analysis of risk factors for anemia in older adults

Variable	Univariate analysis		Multivariate analysis	
	OR (95% CI)	*P*-Value	OR (95% CI)	*P*-Value
Mean age, years	1.077 (1.066–1.088)	< 0.001	1.058 (1.045–1.070)	< 0.001
Women, %	1.318 (1.167–1.488)	< 0.001	1.252 (1.080–1.453)	0.003
Underweight (BMI < 18 kg/m^2^), %	2.322 (1.717–3.139)	< 0.001	2.197 (1.570–3.075)	< 0.001
Social allowance, %	1.311 (1.107–1.552)	0.002	1.194 (0.984–1.449)	0.072
Low household income, %	1.077 (0.961–1.206)	0.201		
Food insecurity	1.104 (0.742–1.644)	0.626		
Live alone, %	1.247 (1.093–1.423)	0.001	1.042 (0.888–1.223)	0.616
Condition, %				
Hypertension	1.139 (1.010–1.284)	0.034	0.902 (0.790–1.029)	0.125
Osteoarthritis	1.187 (1.041–1.353)	0.011	1.109 (0.954–1.290)	0.177
Rheumatoid arthritis	1.628 (1.276–2.078)	< 0.001	1.436 (1.109–1.860)	0.006
DM	1.525 (1.330–1.748)	< 0.001	1.438 (1.248–1.656)	< 0.001
Asthma	0.896 (0.692–1.160)	0.406		
Pulmonary tuberculosis	0.659 (0.149–2.903)	0.581		
Cancer, current	2.360 (1.689–3.298)	< 0.001	2.675 (1.890–3.786)	< 0.001
Hepatitis B	0.670 (0.384–1.169)	0.158		
Hepatitis C	0.787 (0.262–2.369)	0.670		
Liver cirrhosis	1.139 (0.585–2.216)	0.702		
CRF	3.107 (2.734–3.531)	< 0.001	2.557 (2.212–2.955)	< 0.001

*Abbreviations*: *OR* odds ratio, *CI* confidence interval, *BMI* body mass index, *DM* diabetes mellitus, *CVD* cardiovascular disease, *CRF* chronic renal failure

Among comorbid conditions, cardiovascular disease and stroke were analyzed to understand their relationship with anemia (Table 4). Both conditions were significantly related to anemia in the older population.

**Table 4:** Comparison of CVD and stroke of older adults with and without anemia

Variable	Older adults without anemia	Older adults with anemia	*P*-Value
	(*n* = 4,444,812.928)^b^	(*n* = 699,077.287)^b^	
**CVD**	**5.6 ± 0.3**	**8.9 ± 0.8**	**< 0.001**^a^
**Stroke**	**4.0 ± 0.2**	**6.1 ± 0.7**	**< 0.001**^a^

Values are expressed as percentage ± standard error (SE) of the percentage

Abbreviations: *CVD:* Cardiovascular disease

^a^*P*-values were derived from the chi-square test

^b^Weighted population

## Discussion

This study showed a high prevalence of anemia in the population aged 65 years and older, regardless of sex. The prevalence of anemia was found to increase significantly with age, and the trend of prevalence also showed considerable increase within years. Comorbid conditions including being underweight and presence of DM, rheumatoid arthritis, cancer, and CRF were shown to be significant risk factors.

There are few reports on anemia in the older population in Korea. Kim et al. [23] showed that the incidence of anemia in population aged over 60 years in Korea was 7.2%, and most of them were chronically anemic. Han et al. [8] analyzed healthy individuals who underwent routine medical checkup in a single center and showed that mild anemia, defined as having a hemoglobin level between 10.0 g/dL and 12.9 g/dL in men and 10.0 g/dL and 11.9 g/dL in women, is a risk factor for cancer and cardiovascular death in the elderly population. Jeong et al. [24] also showed a high incidence of anemia in the population aged 80 years and more, and most of them were anemic from unknown reasons, which was closely related to malignancy as analyzed by routine medical checkup of laboratory data. These studies are small community-based cross-sectional studies within a selected population and are limited in reflecting the general Korean population. This study analyzed data from the KNHANES, which was elaborately designed and conducted as a government-led nation-wide survey with carefully sampled participants. Therefore, the results of this study potentially represent the current status of older population in Korea.

However, results from this study are limited in their ability to be generalized, because populations with laboratory data displayed significantly varying characteristics compared to populations without laboratory data. The decision to conduct laboratory tests was left entirely to the participants and the decision was made by the family unit in KNHANES; hence, the group with missing laboratory data was not randomly distributed. This seems to contribute to the observed differences.

The occurrence of anemia in older people is not fully understood. Hemoglobin levels in the older population are reportedly lower than the reference values for other population groups. Some reports concluded that this decrease in hemoglobin levels might be one of the consequences of the normal aging process; hence, the criteria for anemia should be reassigned in this population [13, 24–27]. However, many reports also showed that anemia in older individuals is related to the presence of underlying health conditions and is therefore associated with high mortality and morbidity. Furthermore, most people with anemia have been shown to have nutritional deficiency, but the etiology is unknown in one-third of the anemia cases [17, 28]. This unexplained category of anemia includes aging-related clonal hematopoiesis (ARCH), idiopathic cytopenia of undetermined significance (ICUS), and pre-myelodysplastic syndrome (MDS); these conditions are associated with a low but potential risk of hematologic malignancy [19, 20, 29]. Therefore, older people with anemia are recommended to undergo evaluations for etiology analysis [20]. However, there is no guideline or consensus for the range and frequency of the evaluation and management in this population.

The reported prevalence of anemia differs across various studies, and these differences might be attributed to the diversity of the subjects and cohorts of previous studies [4, 17, 23, 24, 27]. Studies conducted in nursing home- and hospital-based populations have shown significantly higher prevalence of anemia and morbidity [3, 10]. Most of these studies seem to show similar findings with regard to the prevalence of anemia in older adults—that is, the prevalence of anemia tends to increase with age, and there is no sex-based difference, with some studies even showing a higher prevalence of anemia among males. In particular, a study in the US showed that in people aged > 85 years, the prevalence of anemia was higher among males [17].

In this study, the prevalence of anemia in the population aged ≥65 years was 14.0%, which is higher than that reported in previous studies, for example, 10.6% in the US [17] and 8.33% in Bang et al’s study [30]. Since study settings and participants enrolled are different, it is not reasonable to compare these studies directly. However, this finding is quite striking because our study was based on populations who were relatively healthy and lived in a secure environment. Moreover, it is expected that the prevalence of anemia would be much higher in those who live in a less secure environment, are admitted in hospitals or social facilities, and experience malnutrition due to financial reasons. Studies of hospitalized patients and people with diseases have shown that anemia is associated with a high risk of complications and poor outcomes [3, 5, 6, 13].

This study showed that older adults with anemia tend to be underweight, which might be related to malnutrition. However, this finding is inconsistent with those of previous studies, showing that being a beneficiary of social allowance or having a low household income is not a risk factor for anemia. Malnutrition-related anemia in the elderly has been reported to be 34–62% of the anemia of known etiology [17, 23, 30, 31]. Most cases of malnutrition-related anemia involve iron, folate, and vitamin B12 deficiency [28]. Among them, iron deficiency is the most common and is usually accompanied by comorbidities, i.e., gastrointestinal tract bleeding, chronic inflammation, among others. Malnutrition also can be induced by chronic diseases, i.e., chronic renal failure, or psychological disorders, i.e., depression and anorexia [28]. Moreover, malnutrition also correlates with the economic status or household financial burden [28, 31]. This nationwide survey did not include any other specific data for malnutrition apart from body weight. Further studies are thus required to clarify the relationship between anemia and malnutrition.

In addition, as shown in Table 1, participants without laboratory data tends to be in worse circumstances; they tend to be older, to have economic problems, and live alone. Prevalence of comorbidities were also higher in those without laboratory data. Laboratory tests were distributed according to the statistically designed manner considering weight and stratifications. However, participants could choose whether to do the laboratory tests or not in the end. Worse circumstances might have hindered from time-consuming, troublesome laboratory tests. It is difficult to find out where these differences came from, but it seems to be obvious that our prevalence of anemia and its burden might be underestimated. If the whole individuals had laboratory data and included in our study, the result would have been quite different.

This study is limited in its ability of the KHNANES data being unable to show a causal relationship between variables. To determine the cause-and-effect relationship between these variables, large well-designed prospective cohort studies are necessary. Another limitation is that the questionnaire items of the KHNANES are being revised every 3 years for economic reasons, making it impossible for researchers to investigate the possible etiologies of anemia in the study population. As the etiologies of anemia seem to be more complicated in older adults than in the younger population, anemia may impose a heavy medical burden on older people in countries such as South Korea.

## Conclusion

In conclusion, this study revealed that age; female sex; being underweight; and presence of rheumatoid arthritis, DM, cancer, and CRF were associated with an increased risk of anemia among older adults in South Korea. Our findings will be very relevant for developing interventions and programs aimed at healthy aging at the individual and societal levels.

## Data Availability

The datasets generated and/or analyzed during the current study are available in the KHNANES repository, https://knhanes.cdc.go.kr/knhanes/eng/index.do.

## Abbreviations

KNHANES: Korea National Health and Nutrition Examination Survey
BMI: Body mass index
DM: Diabetes mellitus
CVD: Cardiovascular disease
CRF: Chronic renal failure
CI: Confidence interval
OR: Odds ratio
ARCH: Anemia includes aging-related clonal hematopoiesis
ICUS: Idiopathic cytopenia of undetermined significance
MDS: Myelodysplastic syndrome
